# Estrogen receptor 2 polymorphism in idiopathic scoliosis

**DOI:** 10.1186/1748-7161-10-S1-P3

**Published:** 2015-01-19

**Authors:** Piotr Janusz, Tomasz Kotwicki, Miroslaw Andrusiewicz, Malgorzata Chmielewska, Malgorzata Kotwicka

**Affiliations:** 1 Spine Disorders Unit, Department of Pediatric Orthopedics and Traumatology, University of Medical Sciences, Poznan, Poland; 2 Department of Cell Biology University of Medical Sciences, Poznan, Poland

## Objectives

In estrogen receptor 2 (ESR2) gene the rs1256120 polymorphism was described to be associated with predisposition to and severity of idiopathic scoliosis (IS) in Chinese population. This observation has not been confirmed in Japanese population. The ESR2 AluI and RasI site polymorphisms were described to present association with breast cancer, rheumatoid arthritis and bone mineral density, however the association with IS has not been evaluated. The purpose of the study was to investigate associations of the ESR2 polymorphisms with either predisposition to or progression of IS in Central European population.

## Material and methods

Case-control study of 248 females with IS and 243 healthy females was performed. Participants underwent clinical, radiological and genetic examination. Three SNPs were studied: AlwNI (C/T rs1256120), AluI (A/G rs4986938) and RasI (A/G rs1256049). Samples were analyzed with polymerase chain reaction followed by restriction fragments length polymorphism analysis (PCR-RFLP). The patients' medical history was evaluated, the Cobb angle was measured and surgery rate established. The patients were divided into three groups according to curve progression velocity: non-progressive IS, slowly progressive IS (progression <1° per month), and rapidly progressive IS (progression >1° per month).

## Results

Neither the genotypes nor allele distribution showed significant differences between IS patients and healthy controls. The results followed Hardy-Weinberg equilibrium (HWE).

There was significant difference neither in genotype nor allele frequency for the non-progressive, slowly progressive, and rapidly progressive idiopathic scoliosis.

In AluI SNP a significant difference in mean Cobb angle between genotypes was found: AA 31.9°±14.2°, AG 43.2°±17.8° and GG 38.9°±19.0°, p=0.0020.

In the patients, divided according to Cobb angle into moderate (<40°) and important (>40°) scoliosis, there was significant difference in genotypes distribution, p=0.001. In this division minor allele frequency (AA) in recessive model of penetration was overrepresented in patients with Cobb angle below 40° with p value 0.0081 and OR (odds ratio) 3.65.

## Conclusions

No association between ESR2 polymorphism and predisposition to IS was found in Caucasian females. None of the previously reported associations could be confirmed, regarding curve severity, progression or surgery rate. AluI site polymorphism may be associated with severity of IS.

